# Psychocultural Mechanisms of the Propensity toward Criminal Homicide: A Multidimensional View of the Culture of Honor

**DOI:** 10.3389/fpsyg.2017.01872

**Published:** 2017-11-01

**Authors:** Monica G. T. C. Souza, Bruno C. Souza, Antonio Roazzi, Edson S. da Silva

**Affiliations:** ^1^Department of Law, Faculdade do Recife, Recife, Brazil; ^2^Department of Management Sciences, Graduate Program in Business Administration, Universidade Federal de Pernambuco, Recife, Brazil; ^3^Department of Psychology, Graduate Program in Cognitive Psychology, Universidade Federal de Pernambuco, Recife, Brazil

**Keywords:** culture of honor, homicide, homicidal honor, criminology, smallest space analysis, Facet Theory

## Abstract

**Introduction:** Theory of the Culture of Honor is one of the few models in criminology specifically geared toward homicide. It proposes that, in certain societies, men must never show weakness and are required to react violently to any perceived threats to their reputation, thereby increasing their probability of committing a homicide. This has been suggested as the main explanation for the high rates of this type of crime in Brazil, particularly in the Northeast. Underlying this explanation there are complex mechanisms and processes that have yet to be clarified.

**Objectives:** The present research aimed to investigate the workings of the possible psychocultural mechanisms underlying the culture of honor and the process through which they might affect the individual propensity toward homicide.

**Methods:** A total of 336 Brazilian adults were assessed regarding a broad range of sociodemographic, psychological, and sociocultural variables, including their attitudes toward homicide. The resulting dataset was analyzed using Smallest Space Analysis and Facet Theory.

**Results:** It seems that certain cultural elements associated to traditional masculinity and enhanced anger tend to promote negative personality traits and increase one’s propensity toward committing homicide.

**Conclusion:** The findings obtained not only confirm the Theory of the Culture of Honor for the propensity toward homicide, but also explicit and clarify some of the psychocultural processes and mechanisms involved, suggesting a new scientific framework.

## Introduction

Homicide is the ultimate form of interpersonal conflict, one that is present in all societies throughout the whole of human history (Durkheim, 1978). Its widespread prevalence in space and time makes it a very relevant social problem with a strong impact on public health, particularly among young males and in developing countries (Dahlberg and Krug, 2002; Pridemore, 2003; Geneva Declaration, 2008; UNODC, 2013; University of Cambridge, 2014).

In Brazil, criminal homicide is a particularly significant problem. According to reports from different sources, the country answers for roughly 10% of the murders on the planet, though it has only some 3% of the population. It is the country with the highest overall incidence of this type of crime, and it is also among the top 10 in terms of occurrences per 100 1000 inhabitants (Geneva Declaration, 2008; UNODC, 2013). This reality produces a very significant social and economic burden upon the nation, especially in the Northeastern Region where the rates are highest (UNODC, 2013). Within that portion of the country, the state of Pernambuco has stood out as being one of the most afflicted with the incidence of lethal violence and, though it seemed to experience a 21% drop in the homicide rates between 2007 and 2014 due to specific public policies to address the issue (SEPLAG-PE, 2014), since 2015 there has been a phenomenal rise in spite of the continuation of said policies (Jornal do Comércio, 2015; TV Jornal, 2016). Indeed, even the intellectual authors of the governmental plan against violence in Pernambuco admit that it has significant flaws (Ratton et al., 2014).

There are multiple scientific models attempting to explain the occurrence of homicide, but the Theory of the Culture of Honor is one oriented specifically toward this type of crime and has produced important interpretations in terms of Social Psychology (Cohen, 1996, 1998; Cohen and Nisbett, 1997). It is based on the notion that certain societies develop a culture that demands that its men never show signs weakness and that they react violently to any threats to their reputation, with “honor” being the central point of their life, making homicide an acceptable or even mandatory form of conflict resolution (Reed, 1982). Some authors have pointed to this sociocultural phenomenon as one of the main causes for the alarmingly high levels of violence in the Brazilian Northeast (Alencar, 2006; Magalhães, 2009). Indeed, there is at least one empirical study done in Pernambuco showing that kind of explanation is more successful than competing theories based on socioeconomic frustration, testosterone levels, moral development, basic moral values, emotional attachment, and decision-making processes (Souza et al., 2011, 2016).

The success of the Theory of the Culture of Honor comes, however, with a series of unanswered questions as to the chain of events involved in the interaction between a collective set of mores and individual actions. Though the works of social psychologists Cohen and Nisbett have described differences between young men from locations with or without a strong culture of honor regarding attitudes toward conflict, propensity to anger, and inclination toward violent confrontation (Cohen and Nisbett, 1997), they do not present clear depiction of a specific model for the mechanisms and processes that interlink an individual’s relatively “internal” psychology to the more “external” sociocultural elements in order to produce violent tendencies.

The present paper aims to contribute to the understanding of the psychocultural workings underlying the culture of honor and its tendency to increase an individual’s propensity toward committing homicide. The approach chosen was to empirically measure one’s internalization of a culture of honor, along with multiple relevant psychological variables (cognition, emotional dynamics, personality) and sociocultural elements (sociodemographics, value assigned to different moral compasses), so as to assess their relationships to each other and to attitudes toward homicide. By using analytical techniques capable of visually expressing the relational structure between multiple variables simultaneously, findings were obtained that can directly inspire a broader scientific model of the phenomenon in question.

A short preliminary version of this work was presented in the 15th Facet Theory Association Conference, 2015, New York, EUA (Souza et al., 2015), the present paper representing a much broader set of theoretical references, empirical results, and theoretical interpretations, as well as the beginnings of a new scientific model.

### Culture of Honor and Homicide

#### The Theory of the Culture of Honor

The theory of the Culture of Honor was initially proposed by John Shelton Reed as a means to explain the fact that, in the late 1800s and early 1900s, some counties in Southern United States had a very high rate of homicides, whilst other counties, sometimes very nearby, did not (Reed, 1982). Based on historical records, he observed that in most of these crimes, victim, and killer knew each other previously and both of them understood the reasons for the killing. Reed also noticed that the communities with the highest historical rates of homicide tended to be those where herding was the main productive activity, as opposed to agriculture. From this he hypothesized that, while in agricultural societies cooperation is a necessity and interpersonal threats to one’s livelihood is relatively low, in societies located in highlands, where the soil is dry and herding is the main source of resources, individual herders are more isolated from each other, and subject to a significant risk of losing their herds to some rival or foe. The rationale is that crops require a significant amount of collective work to be planted, tended, and harvested, plus being relatively difficult to steal in relevant amounts, whereas herds could be looked after by one individual, perhaps with help from the immediate family, but could be stolen in their entirety overnight by an equally small number of people. Given the absence of government to enforce property rights of herd animals in such remote places, in order to avoid becoming a target, an individual had to project the image of being strong, potentially dangerous, and willing to react violently to threats. This means having to always be assertive, aggressive, and defend one’s own standing against any type of challenge. For various biological, evolutionary, and sociocultural reasons, this role traditionally falls upon the adult males. Thus, a Culture of Honor emerges where a man’s reputation is the central point of his work and self-esteem, it being imperative for him to guard it, at all costs, against any possible contention.

In the late 1990s, Richard Nisbett and Dov Cohen not only analyzed social data on homicides that seemed to confirm the theory proposed by Reed (1982), but also did a series of well-designed experimental studies in social psychology where they compared young men from Southern United States (traditionally a region with high levels of the Culture of Honor) to those of Northern United States (considered to have traditionally lower levels of the Culture of Honor) in regards to their propensity toward aggression and violence in response to insults or outrage (Cohen, 1996, 1998; Cohen and Nisbett, 1996, 1997). By employing actors and building specific scenarios of interaction, their investigations showed that, though both Southerners and Northerners were able to respond with violence, the Southern men were significantly more prone to do so, as well as to support the principle of using violence to correct a wrongdoing, the use of corporal punishment, reduced regulation of gun ownership, and so forth. They also found that such a pattern of behavior was something that occurred even in societies where the historical conditions that favor the emergence of the Culture of Honor had significantly changed during the last century or more, indicating the existence of strong sociocultural mechanisms through which such a culture, once existing, is transmitted from one generation to the next by means of tradition.

It is important not to confuse the Culture of Honor, as described by Reed (1982) and Cohen and Nisbett (1997), with the sociocultural phenomenon underlying the so-called “honor killings” (Baker et al., 1999; Awwad, 2007; Abu-Odeh, 2011), as the two are very distinct, even though they both involve “honor” and social standing. The first revolves around a set of mores that compel men to behave violently toward other men in response to perceived physical and/or moral threats, whereas the second refers to societies where males are expected to kill female relatives that are believed to have dishonored the family through what is deemed as immoral behavior of a sexual and/or rebellious nature. The rates of honor killings are difficult to quantify due to the reluctance in certain communities to report or even to classify the crimes as such, but there is strong reason to suspect they have been on the rise for decades (Chester, 2010; Abu-Odeh, 2011), though, worldwide, the vast majority of homicides have men as victims (UNODC, 2013; University of Cambridge, 2014). The focus of the present study is on Culture of Honor Theory, newly applied to a community in Brazil where there is reason to believe that such a model has significant explanatory power.

#### Culture of Honor and Homicides in the Brazilian Northeast

Northeastern Brazil, a region where the geography is that of dry and hot highlands, with a history of herding as one of the main productive activities, seems like an ideal place to test the framework suggested by Reed (1982) and expanded by Cohen (1996, 1998) and Cohen and Nisbett (1996, 1997). Indeed, there have been studies suggesting that a Culture of Honor might be the best explanation for the high rates of homicide recorded in the area (Alencar, 2006; Magalhães, 2009; Souza et al., 2011).

Alencar (2006) interviewed a group of 20 men in Northeastern Brazil that had been convicted for homicide and asked them about the motives for their crime. The group of males aged between 20 and 49 years not only reported revenge for humiliation, threats, and/or aggression as their main reasons to kill, but also expressed the belief that, under the circumstances, their actions were morally justified.

Magalhães (2009) analyzed the influence of “shame” in criminally violent behavior in the Brazilian Northeast. His conclusion was that, in the region in question, dishonor, that is, the loss of social standing and reputation, is not acceptable at all for a man under any circumstances, especially when brought about by others. When faced with such a perspective, the only accepted form of avoiding such a shame is to retaliate the offense with severe and symbolic violence, or, preferably, with death. Such a reaction not only removes the negative shadow that was cast upon the individual when he was subject to the dishonor, but also puts him under a favorable light in the eyes of the community.

An empirical study was done in the state of Pernambuco, in the Brazilian Northeast, to test the efficacy of the Theory of the Culture of Honor, along with theories based on socioeconomic frustration, rationality of the decision-making processes, emotional attachment, testosterone, moral development, moral values (Souza et al., 2011, 2016). A total of 160 adult males (57 convicted for homicide, 63 with other convictions, and 40 without criminal conviction) were submitted to a questionnaire, various psychological tests, and right-hand digit ratio measurements (to estimate testosterone levels). Analysis of the data obtained produced findings indicating that, at least for the study population: (a) homicide is a unique type of crime that doesn’t stem from violence or criminality in general, with violent crimes being more closely associated to non-violent crimes than to homicides; (b) there is usually no characteristic profile for a killer in terms of socioeconomic frustration, decision-making process, attachment, moral values, moral development, or testosterone; (c) the main reason for a homicide is the occurrence of an honor-related motivation, with the motivation for material gain being associated with other crimes (with honor and material motivations shown to be mutually exclusive); and (d) logistic regression models using both honor-related and material gain motivations as predictive variables were capable of correctly identifying more than 80% of the killers in a mixed sample of individuals. Such findings force one to discard all of the theories being tested, save only for the Culture of Honor, which was, therefore, considered as an effective model to explain homicide in the sample studied.

### Culture of Honor and Homicide: Integrating Collective and Individual Elements

#### From the Sociology of Culture to Individual Psychology

Murder is a crime committed by a specific individual or set of individuals toward another individual or set of individuals, therefore, for the Culture of Honor to have an impact on a society’s homicide rate it must necessarily influence internal psychological mechanisms involving volition, cognition, emotion, and behavior. This path from the collective to the individual is recognized in the literature, which mentions material and economic conditions that favor the emergence of certain traditions and institutions associated to social mores that, through feelings of shame, would lead to the activation of anger and rage, thereby producing an increased propensity toward lethal violence as a response to not just physical, but also moral, threats (Reed, 1982; Cohen, 1996, 1998; Cohen and Nisbett, 1996, 1997). However, the specific interplay of these elements, as well as their details and relational structure, have only been described in a broad manner (Guerra, 2009).

#### The Internalization of the Culture of Honor by an Individual

Rodriquez Mosquera et al. (2008) have created an instrument called the Honor Scale which measures the degree to which individuals feel threatened, that is, feel shame and anger, as a consequence of different kinds of attack to their honor. It is comprised of four subscales, classified according to the type of honor involved: Family (reputation of close relatives), Social (integrity and honesty), Masculine (assertiveness and sexual prowess), and Feminine (propriety and sexual modesty). Souza et al. (2013) have extracted from the Honor Scale a fifth index, based on a combination of Masculine Honor (scored positively) and Integrity (scored negatively), named ‘Homicidal Honor,’ which has been positively associated to the propensity toward criminal homicide as approximated by individual experience with homicide (knowledge of authors or victims) and degree of personal condemnation for that type of crime (years of penalty that one would assign to the author of murder under various circumstances).

#### Honor, Society, and Anger

The Culture of Honor refers to the sociocultural and psychological elements that lead the male members of a society to react violently to perceived physical and moral threats (Reed, 1982; Cohen, 1996, 1998; Cohen and Nisbett, 1996, 1997). In terms of individual psychology and from an evolutionary perspective, there are two basic responses to a threat from an adversary: fight (engagement in conflict) or flight (escape or withdrawal from the conflict). Such responses are mediated through emotions that drive behavior, with fear leading to evasion and anger leading to confrontation (Potegal and Novaco, 2010). Therefore, there have to be psychocultural mechanisms in the Culture of Honor that involve anger and rage, as already pointed out. Indeed, culture, society, and emotion have been found to be significantly interwined (Manstead, 2010), particularly with regards to mechanisms of individualism-collectivism, shame, anger, and violence (Gouveia and Clemente, 2000; Potegal and Novaco, 2010; Wranik and Scherer, 2010), these being the key mediators between social mores and criminal violence (Potegal and Novaco, 2010; Wranik and Scherer, 2010). It is, thus, necessary to ponder what role anger and its dynamics play within the Culture of Honor.

#### Individual Temperament

If anger is a key mediator between the cultural and the individual in the context of the Culture of Honor and violent crimes, then the elements associated to the shaping of its internal dynamics, such as emotional regulation and personality, are of importance in the propensity toward homicide. Such elements are also very relevant as to the characterization of individual differences and their role.

Gross and John (2003) created an instrument to measure the degree to which one uses two types of strategy for emotional regulation: Suppression (control of emotional expressions) and Cognitive Reappraisal (changing one’s mood through thought). The first modality has been shown to be associated to poorer mental health and social adjustment, whereas the second was observed to be associated to the opposite. There is clear evidence that such mechanisms are related to the dynamics of anger and to homicide (Matsumoto et al., 2012; Roberton et al., 2014).

Personality is the sum of all the traits that comprise an individual and define his or her uniqueness, therefore, it is, by definition, something that includes one’s emotional dynamics. One of the most famous models is the ‘Big-Five’ (Digman, 1990), which establishes personal psychological traits can be grouped into five major categories:

• Openness to Experience: Intellectual curiosity, creativity and preference for novelty and variety, its opposite may be called Conventionalism;
• Conscientiousness: Tendency to be organized, dependable and self-controlled, its opposite may be called Impulsiveness;
• Extraversion: Assertiveness, sociability, talkativeness and expansiveness, its opposite being Introversion;
• Agreeableness: Tendency to be compassionate, altruistic and cooperative toward others, its opposite may be called Misanthropy;
• Neuroticism: Tendency toward negative emotions, emotional instability and maladjustment, its opposite being Stability.

A meta-analysis of over 60 studies suggests that individuals with a high level of Neuroticism, as well as low levels of Agreeableness and Conscientiousness, are more likely to engage in antisocial behavior, including violent crimes and homicide, though there may be complex interactions with gender and sociocultural variables (Miller and Lynam, 2001).

#### Moral Compasses

Individual morality, values, and attitudes are guided by several directives provided by sources such as law, religion, family, customs, and personal will, each of which could be labeled a “Moral Compass,” with different impacts on the Culture of Honor according to both individual traits and the type of society one is inserted in Reed (1982), Cohen (1996, 1998), and Cohen and Nisbett (1996, 1997). Thus, it is relevant to go beyond the mere acknowledgment that there are societal mechanisms and processes that somehow jointly produce the Culture of Honor and an increased propensity toward homicide. It is required that the researchers in the field strive for an understanding of the specific ways in which each Compass might relate to a greater or lesser degree of said culture, to specific aspects of it, and to an individual’s internal psychology. Preferably, such an understanding is to be grounded on systematic empirical observations, rather than solely on speculation.

#### Hyperculture

The Digital Revolution is a term that refers to developments in information and communication technologies (ICTs) that became practically omnipresent in daily experience from the last decade of the 20th Century onward. Such developments have radically transformed the productive forces of society, changing its economy, politics, and culture in many ways, leading to novel ways of thinking and acting (Souza et al., 2010, 2012; Souza and Rangel, 2015).

The Cognitive Mediation Networks Theory (CMNT) is a scientific model of human cognition that aims to explain the complex interactions between society, technology, and thought. When applied to the Digital Revolution, it points to the emergence of a Hyperculture consisting of the forms of thinking and acting linked to the ICTs and the sociocultural structures created around them, with impacts regarding:

• Cognition: Inclination toward logical-numerical, abstract and visual-spatial reasoning with recombinant/fragmented thinking, leading to increased speed-of-processing, multitasking, emotional-intuitive (ludic) creativity, and overall cognitive performance;
• Personality: Greater tendency toward Openness to Experience, Conscientiousness, and Stability (low Neuroticism), as well as to a high degree of intellectualism, and to assign more importance to knowledge, success, maturity, and aesthetics as personal values;
• Social Interactions: Increased propensity toward communication, sociability, collaboration, and leadership;
• Relationship with Technology: Frequent, intense and broad use of digital technologies in general, along with a high level of mastery in their daily use;
• Work Life: Greater levels of professional updating, continued education, professional versatility, and entrepreneurism, as well as a tendency to assign more professional importance to knowledge, individual competence and the mastery of technology, leading to greater economic success.

There is strong empirical evidence of such positive impacts (Souza et al., 2010, 2012; Souza and Rangel, 2015), whereas concerns regarding potential negative effects of ICTs in terms of diminishing the capacity for concentration and contemplation (Carr, 2010) or even *per se* promoting obsessive behavior, addiction and psychopathologies (Aboujaoude, 2011) seem to be either entirely baseless or grossly overstated.

The cognitive, social and personality traits linked to the Hyperculture, as well as its positive association to professional life (Souza et al., 2012; Souza and Rangel, 2015), all seem to indicate that this new form of thinking and acting relates to social adjustment and psychological well-being in general. There is also the fact that society and culture have been shown to significantly influence emotional dynamics and expression, especially regarding anger (Gouveia and Clemente, 2000; Potegal and Novaco, 2010; Wranik and Scherer, 2010). This suggests that the Hyperculture favors an enhanced tolerance of diversity, social interaction, and dialog, promoting communication, mutual understanding, and peaceful means of conflict resolution, thereby counteracting the mechanisms and processes through which it is considered that the Culture of Honor promotes homicide (Souza et al., 2014).

### The Need for a Multidimensional Approach

Even when one focuses on a specific theory such as the Culture of Honor, criminal homicide reveals itself to be a complex phenomenon involving a wide diversity of variables with many possible interactions, including a great amount of covariation, all of which can cause confusion when it comes to data analysis and interpretation using traditional techniques. It appears, therefore, that a study of the propensity toward homicide encompassing a broad set of psychological and sociocultural variables can substantially benefit from the use of sophisticated multivariate analysis techniques.

### Study Goals

The present study aimed to investigate the relationships between the different components of the culture of honor and an individual’s experience and attitudes toward criminal homicide, with the identification of the roles of internal psychological variables regarding the dynamics of anger, emotional regulation, and personality, as well as the psychosocial elements of Hyperculture and the value assigned to different moral compasses. The main goal was to use SSA and Facet Theory to observe the relational structure of these multiple variables and glimpse the workings of the psychocultural mechanisms underlying an individual propensity toward homicide in light of the Theory of the Culture of Honor.

## Materials and Methods

### Subjects

A total of 336 adult Brazilian subjects from the Metropolitan Region of Recife, 169 men and 167 women, with the mean age of 34.6 years (*SD* = 11.13), 69.6% of which with up to intermediate level education, 23.5% with higher education and 6.8% with a graduate degree.

### Instruments

• One form containing 30 questions regarding:
  ○ Sociodemographics;
  ○ Degree of experience with victims and authors of homicides;
  ○ Degree of condemnation of homicides (years of penalty assigned);
  ○ Importance assigned to law, religion, family, customs, and personal will;
  ○ Recent experience with anger (intensity, duration, rumination, frequency).
• Hypercultural Index, Digital Experience and Digital Precocity (Souza et al., 2012);
• Portuguese language version of the Ten Item Personality Inventory (Gosling et al., 2003);
• Portuguese language version of the Emotional Regulation Questionnaire (Gross and John, 2003);
• Portuguese language version of the Honor Scale (Rodriquez Mosquera et al., 2008).

### Procedure

A total of 48 law students from a private higher education institution Recife, Pernambuco, Brazil, approached the subjects in the streets of the Metropolitan Area of Recife, invited them to participate, and applied the instruments to those who accepted, which was done in various locations according to convenience, with the interviewer registering all the responses in previously printed spreadsheets. Each student interviewed a total of seven subjects. Those participants who eventually failed to answer to all the questions and/or to do so in the appropriate format had their data discarded and another subject was selected to replace him/her.

### Analysis

#### SSA and Facet Theory

Facet Theory is a powerful mathematical approach that allows one to juxtapose theory and observations in complex phenomena by means of a meaningful visual holistic representation of the relationships within a dataset. It is based on a particular form of Multidimensional Scaling, called the Smallest Space Analysis (SSA), where the association between two variables is inversely expressed as the distance between them in a graph (the stronger the association, the smaller the distance). Groupings of variables (as in Cluster Analysis), as well as the identification of latent dimensions (as in Factor Analysis), are achieved by means of the geometrical partitioning of the graph into regions that are interpreted as both clusters and constructs. The technique is robust enough to deal with practically any type of data and measure of association between variables. It is no exaggeration to state that it may be one of the most sophisticated and far-reaching forms of multivariate data analysis in existence (Guttman and Greenbaum, 1998; Levy, 2005; de Leeuw and Mair, 2009; Borg et al., 2012). For all of these reasons, SSA and Facet Theory were chosen as the main form of data analysis for the present investigation.

#### Variables, Indexes, and Scores

Some of the elements analyzed were assessed as direct measurements of a variable as recorded by the instruments used, including:

• Dynamics of Anger: The Intensity (1–9 Likert scale), Duration (hours) and level of Rumination (1–9 Likert scale) of the most recent episode of anger, along with the Frequency of episodes (one divided by the time since the last episode);
• Moral Compasses: The importance assigned to Law, Religion, Customs, Personal Will and Family when making a decision, all measured on a 1–5 ordinal scale.

The psychological measures from the standardized tests had their scores all calculated according to the official instructions, this including:

• Culture of Honor: The four components of the Honor Scale, that is, Masculine Honor, Feminine Honor, Social Honor, and Family Honor, all on a 1–7 scale (Rodriquez Mosquera et al., 2008);
• Personality: Scores for the negative versions of the Big Five personality dimensions, that is, Convencionalism, Impulsiveness, Introversion, Misanthropy and Neuroticism, all measured on a 1–7 Likert scale (Gosling et al., 2003);
• Emotional Regulation: Scores for the levels of Cognitive Reappraisal and Emotional Suppression, all on a 1–7 scale (Gross and John, 2003);
• Hyperculturality: The Hypercultural Index (0–1 interval scale), along with Digital Experience (duration of one’s experience with ICTs in years) and Digital Precocity (one divided by the age when one began one’s experiences with ICTs), all three on interval scales (Souza et al., 2012).

Three aspects of one’s relation with homicides were calculated through scores that were created specifically for the present study, these being:

• Experience with Homicides: Close or cursory personal contact with victims homicides, plus personally knowing the authors of a homicide, each measured on a 0–1 dichotomous scale, producing a score expressed as a 0–3 ordinal scale with a Cronbach Alpha of 0.64;
• Tolerance of Homicide: The mean penalty, in years, that one would assign to the author of a homicide under six circumstances (murder of wife due to unfaithfulness, murder of wife’s lover, murder of person who disrespected him, murder of man in order to rob him, murder of another man during a fight, murder of a criminal by another criminal) subtracted from the value of 30 years (maximum penalty in Brazil), generating a 0–30 interval scale with a Cronbach Alpha of 0.88;
• Homicidal Honor: Masculine Honor plus the reverse of Social Honor, as measured by the Honor Scale, an index measured on a 1–9 scale that was positively correlated to Experience with Homicides and Tolerance of Homicides.

All of these dimensions were measured on at least an ordinal scale where equal intervals can be assumed, so that 1-Pearson r can validly be considered as a measure of the distance between them in a SSA.

## Results

### Smallest Space Analysis

**FIGURE 1:**
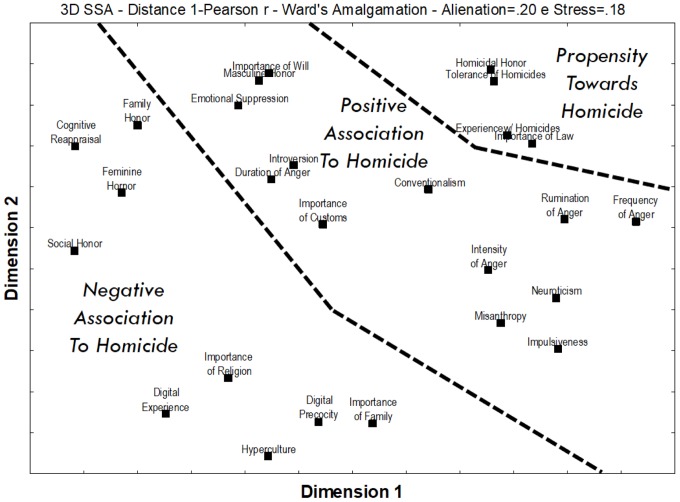
SSA of the main variables related to the propensity toward homicide.

**Figure 1** shows the SSA diagram for the variables in the study using as a metric the 1-Pearson r distance, and Ward’s amalgamation schedule, yielding a Stress value of 0.18, which very much acceptable for an analysis with 26 variables.

The resulting diagram shows a structure that can be clearly divided into three axial partitions.

Tolerance of Homicides, Experience with Homicides and Homicidal Honor were all placed together in the same region of the graph, defining a partition that may be regarded as Propensity toward Homicide. Given its position in the SSA, Importance of Law was also included in this partition.

All the variables related to anger (Intensity, Rumination, Duration, and Frequency) and personality (Neuroticism, Introversion, Misanthropy, Impulsiveness, and Conventionalism), were in a region adjacent to the Propensity toward Homicide. In the same vicinity were also Importance of Personal Will, Importance of Customs, Emotional Suppression, and Masculine Honor. Due to their proximity to the Propensity toward Homicide, which is indicative of a positive association, one may label this partition as Positive Association to Homicide.

In the remaining portion of the SSA plot are Hyperculture, Digital Experience (years of experience with the regular use of computers), and Digital Precocity (reverse of the age in which one began to have regular contact with computers), as well as the Importance of Family and Religion, Cognitive Reappraisal, Family Honor, Social Honor, and Feminine Honor. Due to its distance from Propensity toward Homicide, this partition encompasses elements that seem to be negatively associated to homicides, so it may be called Negative Association to Homicide.

### Comparing Men and Women

**Table 1** compares the men and women in the sample with regards to honor, emotional regulation, and relationship with homicide.

**Table 1:** Comparison between men and women as to honor, emotional regulation, and homicide.

Psychocultural element		Men (*n* = 169)		Women (*n* = 167)		Mann–Whitney *U* (*p*-value)
			
		Mean	*SD*	Mean	*SD*	
Honor (Likert 1–9)	Family	7.71	1.261	7.65	1.292	0.74
	Social	7.62	1.269	7.87	1.083	0.06
	Masculine	6.58	1.462	6.33	1.32	0.01
	Feminine	4.59	2.027	6.00	2.034	<0.01
Emotional regulation (Likert 1–7)	Reappraisal	4.96	1.353	5.26	1.291	0.05
	Suppression	4.30	1.473	3.99	1.491	0.04
Homicide	Experience (Ordinal 0–3)	1.24	0.961	1.17	0.948	0.48
	Penalty assigned (years)	15.70	8.257	19.27	7.937	<0.01
	Homicidal honor (1–9)	4.48	0.785	4.23	0.729	<0.01


Men were found to have statistically more Masculine Honor and Emotional Suppression, as well as more Homicidal Honor and less severe penalty assigned to homicides. Women were found to have statistically more Feminine Honor and Cognitive Reappraisal, and marginally more Social Honor.

## Discussion

### A Proxy for the Propensity toward Homicide

The presence of Homicidal Honor, Tolerance for Homicides and Experience with Homicides in close proximity to each other in the same region of the SSA diagram indicates that these three variables have something in common between them, the most obvious element here being homicide itself. Indeed, it is quite reasonable to assume that individuals with a high level of all three is more likely to commit a homicide than one who has low levels of the same. The rationale here is that Homicidal Honor would lead to an inclination toward killing as a way of dealing with conflict, Tolerance for Homicides would indicate the acceptance of homicide as an alternative of action, and Experience with Homicides would be a source of habituation, and, therefore, a level of indifference, toward this sort of crime.

*Prima fa*cie, the presence of the Importance of Law in this partition seems strange, as homicide is universally illegal. However, it is known that in places with intense levels of the culture of honor, such as the southern portion of the United States, following rules of manners, politeness and propriety, which are frequently a significant part of the traditional charm and hospitality of such places, is a way to reduce the probability of fatal conflict, something which may include abiding to the law in general (Reed, 1982; Cohen, 1996, 1998). Also, it may be that, within the context of a culture of honor, murdering someone is considered as a valid form of resolution for conflicts involving matters of honor, thus, it would not be seen as a violation of the law, but rather an obeisance to it or to some higher principle (e.g., “natural law,” “divine right,” “moral principle”).

### Factors with Positive Association to Homicide

Within the partition of Positive Association to Homicide in the SSA were the strength of Masculine Honor (assertiveness), the Importance of Personal Will (“alpha male”), the Importance of Customs (tradition and propriety), and Emotional Suppression (no display of fear, anxiety, or pity), all of which describe fairly well the moral imperatives for men in the culture of honor (Reed, 1982; Cohen, 1996, 1998).

Also within Positive Association to Homicide were all of the variables related to the dynamics of anger and all the negative dimensions of personality, both types appearing in the SSA as being fairly intermixed with each other, indicating that these emotional and dispositional elements are positively related to each other and the propensity to commit murder. This is very much in agreement not only with the literature on the role of emotion and personality in violent crimes (Miller and Lynam, 2001; Matsumoto et al., 2012; Roberton et al., 2014), but also with the phenomena described in the Theory of the Culture of Honor (Reed, 1982) and in the experimental findings regarding the responses to conflict, insult and outrage in such cultures (Cohen and Nisbett, 1996, 1997).

The finding of a single partition for both the aforementioned sets of elements suggests that the mores of the culture of honor in terms of how one must react violently to insult and conflict have psychological repercussions upon the individual in terms of personality and the dynamics of anger, thereby promoting violent and potentially lethal behavior.

### Factors with Negative Association to Homicide

Within the partition labeled Negative Association to Homicide were Social Honor, Feminine Honor, and Family Honor, along with Cognitive Reappraisal. This suggests that such values are associated to adopting emotional regulation strategies based on internal resolution, rather than the mere controlling of external manifestations, with this combination tending to reduce the predisposition toward lethal violence.

Also inside Negative Association to Homicide were Hyperculture, Digital Precocity, and Digital Experience. They presented themselves fairly close to each other, which is in agreement with the predictions and findings of the CMNT that the greater the amount of interaction with the digital culture and the earlier this process begins, the greater will tend to be an individual’s internalization of the Hyperculture (Souza et al., 2012). Likewise, their opposition to the tendency to commit homicide is in agreement with the theoretical expectation that hypercultural values favor peace and understanding (Souza et al., 2014).

Interestingly, the Importance of Religion and the Importance of Family as moral compasses were yet also present in Negative Association to Homicide, and situated very closely to the variables related to digital culture. It may be the case that religion tends to value peace and non-violence, while family favors the acceptance, protection and safety of its members, all of these being things that would reduce the propensity toward lethal violence. Such values would also be in alignment with those of the Digital Age and Hyperculture (Souza et al., 2014).

### Overview of the Mechanisms and Processes Observed

Tolerance of Homicides, Homicidal Honor, and Experience with Homicides are all associated to one another and proxies of an individual’s tendency to commit murder, i.e., his or her Propensity toward Homicide, the central point of this investigation.

With positive association to Propensity toward Homicide, one finds:

• Importance of Personal Will, Importance of Customs and Masculine Honor, all of which would constitute Aggressive Values;
• Intensity, Rumination, Duration and Frequency of Anger, all of which are the parameters for the dynamics of Rage;
• Neuroticism, Misanthropy, Conventionalism, Impulsiveness and Introversion, which are the opposite of, respectively, Stability, Agreeableness, Openness, Conscientiousness and Extraversion, thereby comprising Negative Personality;
• Emotional Suppression.

With negative association with Propensity toward Homicide, one finds:

• Social Honor, Feminine Honor, Family Honor, Importance of Religion and Importance of Family, all of which would constitute Peaceful Values;
• Digital Experience and Digital Precocity, that lead to the development of an individual’s Hyperculture;
• Cognitive Reappraisal.

There was a positive association between Propensity toward Homicide and the Importance of Law, the latter probably as a consequence of the former because it is a means of preventing unnecessary conflicts and/or an expression of the belief that honor-motivated homicide is righteous.

**FIGURE 2:**
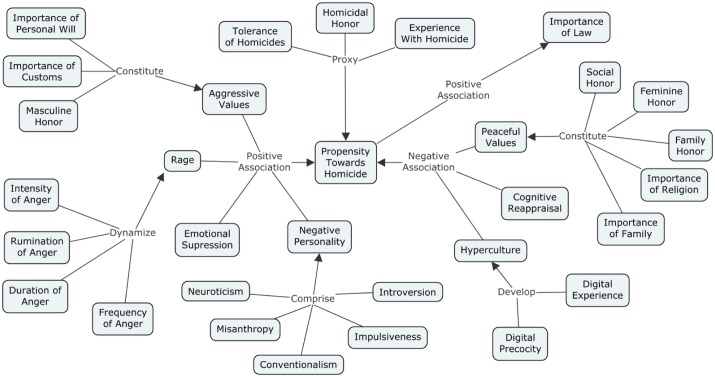
Concept map of the empirical findings of the present study.

**Figure 2** presents a concept map that summarizes the elements that have positive or negative associations to the Propensity toward Homicide, expressing a synthesis of the main empirical findings of the study.

In light of the Theory of the Culture of Honor and the additional frameworks regarding cognition, emotion, moral values and personality that have been approached in the present paper, it is possible to interpret such observations so as to suggest a model for the phenomenon under consideration.

It would appear that a culture that values male assertiveness tends to produce moral values favoring aggression and violence as a means of conflict resolution, with an emotional dynamic where one unsuccessfully attempts to suppress anger, leading to rage that manifests itself in the form of negative personality. In those with a high degree of internalization of such mores, a relevant social occurrence triggering this ensemble of mechanisms, such as an insult or confrontation, tends to favor the arising of an intention to kill, both as an emotional reaction and as an obeisance to a code of conduct. This, in turn, tends to eventually translate into actual homicidal behavior.

Inversely, the internalization of standards of honor regarding demureness, propriety and integrity, particularly stemming from family and religion, favor moral values that favor peaceful forms of conflict resolution, mediated by the more effective mode of emotional regulation that is Cognitive Reappraisal. The Digital revolution and the emergence of the Hyperculture, which are associated to strong social interaction and collaboration, seem to likewise favor peaceful forms of resolving conflicts. This tends to reduce the probability of one engaging in lethal violence.

If the mechanisms and processes that promote homicide surpass those that inhibit it, apparently a heightened valuing of the law emerges, most likely as a form of moderating what would be otherwise an endless streak of lethal violence.

**FIGURE 3:**
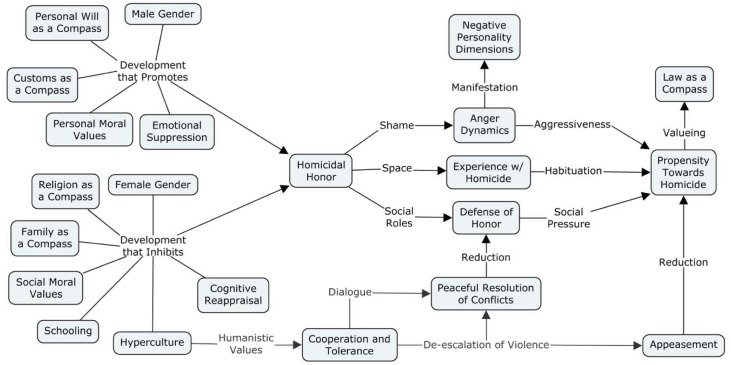
Concept map of the mechanisms and processes suggested by the empirical findings.

A summary of such a model can be expressed by the concept map in **Figure 3**.

This view constitutes a framework that goes beyond the scope of the Theory of the Culture of Honor, describing both psychological and sociocultural dimensions of the phenomenon of homicide.

### Men vs. Women

The differences observed between the genders as to the intensity of the different types of honor were consistent with the traditional social roles prescribed by the culture of honor (Reed, 1982), i.e., that men should be assertive (Masculine Honor) and women should be demure (Feminine Honor) and virtuous (Social Honor). This seems to have translated into males being more prone than females to adopting Emotional Suppression as a means of emotional regulation, whereas females were observed to be more prone to Cognitive Reappraisal than males.

From the Theory of the Culture of Honor (Reed, 1982; Cohen, 1996, 1998), as well as the literature on emotion (Miller and Lynam, 2001; Matsumoto et al., 2012; Roberton et al., 2014), one would expect the observed difference between men and women to lead to a greater propensity toward homicide, which was the case as measured by the Condemnation of Homicides and Homicidal Honor (Experience with Homicides showed no difference, due to the fact that it depends on one’s location rather than gender).

These findings confirm not only the psychocultural mechanisms and processes emerging from the present investigation, but also how they function for men and women as a consequence of the traditional social roles for each gender. This appears to be a viable explanation for the great predominance of male authors and victims of homicide that is observed worldwide (UNODC, 2013).

## Conclusion

The present study aimed to empirically explore the relational structure of various psychological and sociocultural elements involved in an individual’s tendency toward committing murder in light of the Theory of the Culture of Honor.

The findings obtained by means of using proxies for the propensity toward homicide along with adopting a multidimensional analytical approach (SSA and Facet Theory) and comparisons between genders suggest that:

• A culture of honor based on the need to be assertive is associated to a traditional masculinity and enhanced anger which is poorly managed, leading to negative personality traits, all of which tends to increase one’s propensity toward committing homicide;
• The internalization of the culture of the Digital Age, along with valuing family and religion as moral compasses, improved emotional regulation, and a non-aggressive sense of honor seem to disrupt the mechanisms and processes that promote homicide, leading to a reduced propensity toward that type of crime;
• The specific gender roles assigned by the culture of honor tend to put into motion the above psychological and sociocultural elements so as to make men much more prone to homicide than women.

It seems that present study appears to not only confirm the Theory of the Culture of Honor for the propensity toward homicide, but also explicit some of the psychocultural processes and mechanisms involved, thereby producing the beginnings of a new scientific model of the phenomenon.

Future studies on this topic should study a broader and more diversified sample of individuals, as well as additional variables of interest, such as measures of basic moral values and nuances of one’s attitudes toward homicide.

## Ethics Statement

As established by the ethical guidelines for scientific research with human subjects in Article 1, Subsection V, of Resolution no. 510 from the Brazilian National Council on Health, the present study was exempt from registration or evaluation from the country’s Council of Ethics in Research and National Council of Ethics in Research due to the fact that no identification of subjects was registered or even asked for, no experimental intervention was done on the participants that might generate any risks above those of daily life, and absolutely no form of diagnosis or counselling was offered either as a consequence of the responses or any other basis. In spite of that, such official registration was done through the Committee of Ethics in Research of the Federal University of Pernambuco (CAAE: 18036213.0.0000.5208) and a favorable decision was obtained (DECISION: 930.677). In accordance to international principles regarding research ethics, the participation in the present study was fully informed and strictly voluntary.

## Author Contributions

All authors (MGTCS, BCS, AR, and ESS) equally contributed to all the following issues of the research: conception and design of the work; acquisition, analysis, and interpretation of data; drafting the manuscript and critically revising it; final approval of the version to be published; agreed to be accountable for all aspects of the work in ensuring that questions related to the accuracy or integrity of any part of the work are appropriately investigated and resolved.

## Conflict of Interest Statement

The authors declare that the research was conducted in the absence of any commercial or financial relationships that could be construed as a potential conflict of interest.
